# Canonical signaling by TGF family members in mesenchymal stromal cells is dispensable for hematopoietic niche maintenance under basal and stress conditions

**DOI:** 10.1371/journal.pone.0233751

**Published:** 2020-05-29

**Authors:** Joseph Ryan Krambs, Grazia Abou Ezzi, Juo-Chin Yao, Daniel C. Link

**Affiliations:** Division of Oncology, Department of Medicine, Washington University School of Medicine, Saint Louis, MO, United States of America; Augusta University, UNITED STATES

## Abstract

Mesenchymal stromal cells are an important component of the bone marrow hematopoietic niche. Prior studies showed that signaling from members of the transforming growth factor (TGF) superfamily in mesenchymal stromal cells is required for normal niche development. Here, we assessed the impact of TGF family signaling on niche maintenance and stress responses by deleting *Smad4* in mesenchymal stromal cells at birth, thereby abrogating canonical TGF signaling. No alteration in the number or spatial organization of CXCL12-abundant reticular (CAR) cells, osteoblasts, or adipocytes was observed in *Osx-Cre*, *Smad4*^*fl/fl*^ mice, and expression of key niche factors was normal. Basal hematopoiesis and stress erythropoiesis responses to acute hemolytic anemia were normal. TGF-β potently inhibits stromal CXCL12 expression in vitro; however, G-CSF induced decreases in bone marrow CXCL12 expression and subsequent hematopoietic stem/progenitor cell mobilization were normal in *Osx-Cre*, *Tgfbr2*^*fl/fl*^ mice, in which all TGF-β signaling in mesenchymal stromal is lost. Finally, although a prior study showed that TGF-β enhances recovery from myeloablative therapy, hematopoietic recovery following single or multiple doses of 5-flurauracil were normal in *Osx-Cre*, *Tgfbr2*^*fl/fl*^ mice. Collectively, these data suggest that TGF family member signaling in mesenchymal stromal cells is dispensable for hematopoietic niche maintenance under basal and stress conditions.

## Introduction

The bone marrow contains a complex dynamic population of stromal and hematopoietic cells that together generate a unique microenvironment, or niche, to support hematopoiesis. Mesenchymal stromal cells (MSCs) are an important component of the bone marrow hematopoietic niche and include CXCL12-abundant reticular (CAR) cells, adipocytes, osteolineage cells, arteriolar pericytes, and mesenchymal stem cells, all of which have been implicated in hematopoietic stem/progenitor cell (HSPC) maintenance.[1–7] The signals that regulate MSCs and their impact on hematopoiesis are not well characterized.

There is evidence that TGF-β signaling regulates MSCs in the bone marrow. TGF-β has complex stage-specific effects on bone marrow MSCs. It stimulates osteoprogenitor proliferation and induces mesenchymal stem cell migration, while inhibiting terminal osteoblast differentiation.[8] *In vitro* modeling of the interaction between TGF-β and bone marrow MSCs reveal its potential to negatively regulate adipocyte and osteoblast differentiation while promoting osteoblast progenitor proliferation.[9–11] In addition, genetic abrogation of TGF-β signaling in mesenchymal progenitor cells during development results in impaired osteoblast differentiation and a marked expansion of CAR cells and bone marrow adiposity.[12,13] These stromal alterations are associated with a shift in hematopoiesis from lymphopoiesis to myelopoiesis.[12] In contrast, abrogation of TGF-β signaling in mesenchymal progenitor cells at birth (using a doxycycline-inducible *Osx-Cre* transgene) resulted in no discernable alterations in the niche or basal hematopoiesis. Thus, TGF-β signaling in mesenchymal cells during development is required for the establishment of a normal hematopoietic niche but is dispensable for niche maintenance in adults under steady-state conditions.

There also is evidence that other members of the TGF family of cytokines may contribute to the development, maintenance, and/or function of MSCs in the bone marrow. The TGF superfamily consists of approximately 45 ligands divided into four subgroups: TGF-βs, decapentaplegic-Vg-related (DVR), activins/inhibins, and other distant TGF members.[14,15] The DVR subgroup consists of bone morphogenetic proteins (BMPs) and growth differentiation factors (GDFs) which play a critical role in skeletal patterning and soft and hard tissue development.[8,16–20] Deletion of *Bmpr1a* (Alk3) in hematopoietic and stromal cells using *Mx1-Cre*, which targets osteoblast lineage cells, is associated with an increase in N-cadherin positive osteoblasts and a modest increase in HSCs, suggesting that BMP signaling in stromal cells may negatively regulate the stem cell niche.[21] Multiple groups have also shown that inhibition of activin signaling by treating with an activin receptor 2 alpha (ACVR2a) ligand trap stimulates erythropoiesis *in vivo*.[22–25] Indeed, clinical trials have demonstrated improvements in anemia in patients with myelodysplastic syndrome treated with sotatercept, a ACVR2a antagonist.[23,26] Although ACVR2a signaling in erythroid progenitors contributes to this effect,[22,24] two groups showed that inhibition of ACVR2a signaling in bone marrow stromal cells also stimulates erythropoiesis.[23,27]

Myelosuppressive therapy induces marked alterations in the bone marrow microenvironment that includes an expansion of osteolineage cells and adipocytes, which have been linked to hematopoietic recovery.[28,29] Relevant to this study, myelosuppressive chemotherapy activates TGF-β in the bone marrow, and inhibition of TGF-β signaling enhances recovery from chemotherapy.[30] Whether TGF-β or other TGF family member signaling in mesenchymal cells contributes to stromal and hematopoietic responses to myeloablative therapy or other stressors is an open and clinically relevant question. Of note, recent single cell RNA sequencing of bone marrow stromal cells shows that *Tgfbr1*, *Tfgbr2*, *Tgfbr3*, and *Smad 4* are expressed in osteolineage cells and perivascular stromal cells.[31]

TGF family ligands bind to their cognate type I and type II serine/threonine kinase receptors to phosphorylate and active pathway-restricted SMADs (R-SMADs), which in turn complex with SMAD4 to active target genes. Thus, SMAD4 is required for all canonical TGF family signaling. Here, we show that deletion of *Smad4* in bone marrow MSCs at birth results in no discernible alteration in the bone marrow hematopoietic niche. Indeed, basal and stress hematopoiesis are normal. These data suggest that canonical TGF-β family signaling is not required for hematopoietic niche maintenance or niche response to certain hematopoietic stressors.

## Results

### Post-natal loss of *Smad4* in MSCs does not alter the bone marrow stromal microenvironment

**Fig 1:**
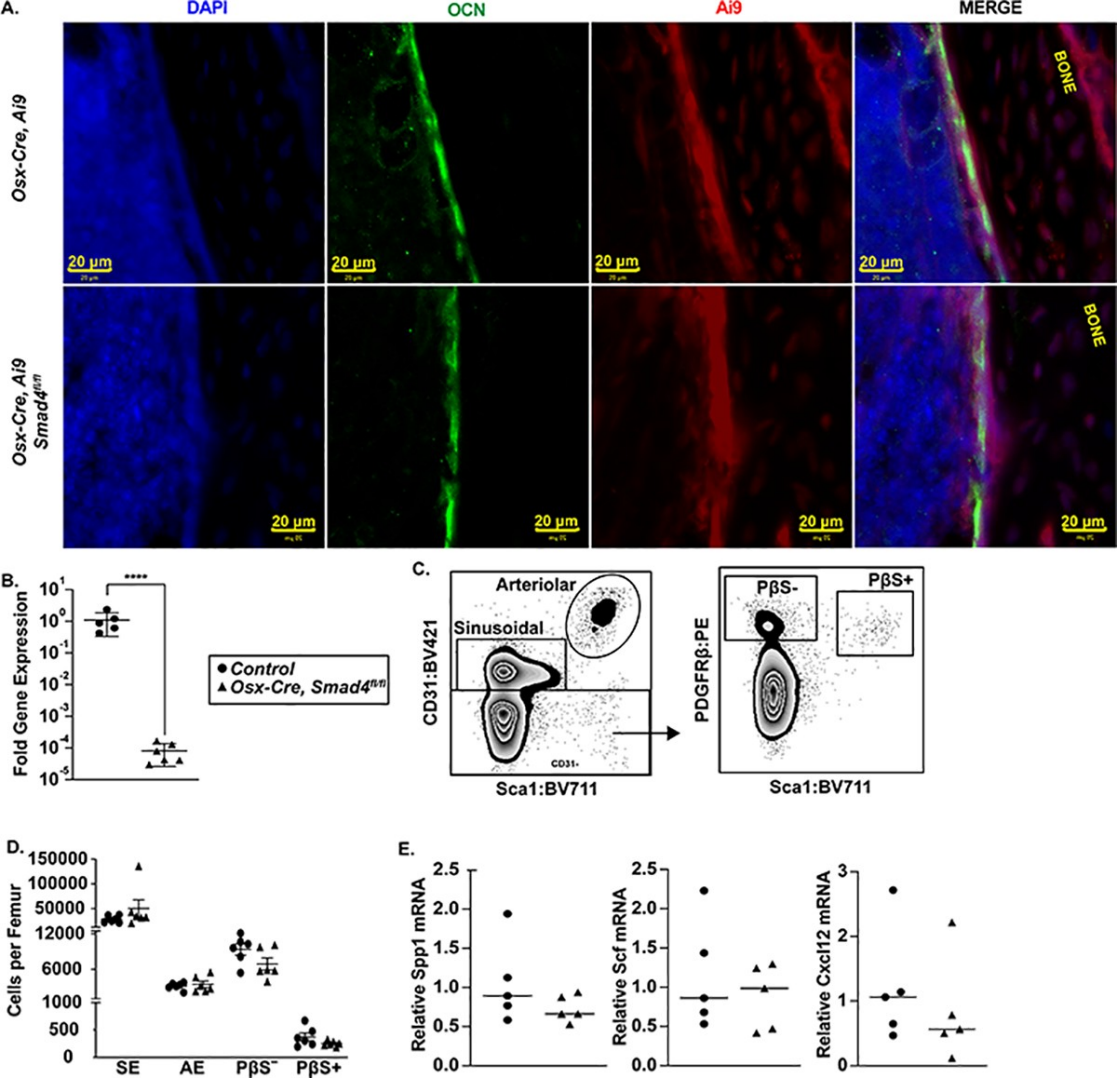
Mesenchymal stromal cell number and organization are normal in *Osx-Cre Smad4*^*fl/fl*^ mice. (A) Representative photomicrographs of femur sections from *Osx-Cre Ai9* or *Osx-Cre*, *Ai9*, *Smad4*^*fl/fl*^ mice showing DAPI stained nuclei (blue), osteocalcin (OCN) expressing mature osteoblasts (green), and tdTomato^+^ mesenchymal stromal cells (red). (B) Relative *Smad4* mRNA expression of sorted tdTomato^+^ CD45^-^ Ter119^-^ stromal cells normalized to *β-*actin mRNA. (C) Representative flow plots showing the gating strategy used to identify CD31^+^ Sca1^–^ sinusoidal endothelial cells (SE) and CD31^+^ Sca1^+^ arteriolar endothelial cells (AE, left panel); data are gated on CD45^–^Ter119^–^ cells. The right panel shows PDGFRβ^+^ Sca1^–^ CD31^–^ mesenchymal stromal (PβS^–^) cells and PDGFRβ^+^ Sca1^+^ (PβS^+^) mesenchymal stem cells. (D) Number of SE, AE, PβS^–^, and PβS^+^ cells per femur is shown for *Osx-Cre* (control) or *Osx-Cre Smad4*^*fl/fl*^ mice. (E) Relative mRNA expression in whole bone marrow compared to *β-actin* mRNA of the indicated gene. Data represent the mean ± SEM. ****P < 0.0001 by unpaired student t-test.

To investigate the role of canonical TGF family member signaling in bone marrow MSCs to hematopoietic niche maintenance, we deleted *Smad4* in mesenchymal cells using a doxycycline-repressible *Sp7* (osterix)-*Cre* transgene (*Osx-Cre*). Prior studies have shown that *Osx-Cre* targets the majority of MSCs in the bone marrow, including osteoblasts, adipocytes, pericytes, and CXCL12-abundant reticular (CAR) cells, but not endothelial cells or hematopoietic cells.[5,32,33] We previously reported that constitutive activation of *Osx-Cre*, by maintaining *Osx-Cre*, *Smad4*^*fl/fl*^ mice off of doxycycline throughout embryonic development, results in a loss of osteoblasts, a marked increase in adiposity, and severe runting.[12] Thus, in this study, we activated the *Osx-Cre* transgene post-natally by removing doxycycline 1–2 days after birth. To confirm targeting, we performed lineage mapping studies on 6-to-8-week-old *Osx-Cre*, *ROSA26*^*Ai9/+*^ (*Ai9*), *Smad4*^*fl/fl*^ mice, in which tdTomato expression is induced in cells that have undergone *Cre*-mediated recombination. As expected, in control *Osx-Cre*, *Ai9* mice tdTomato^+^ perivascular CAR cells and osteocalcin+ endosteal osteoblasts were observed; a similar pattern was observed in *Osx-Cre*, Ai9, *Smad4*^*fl/fl*^ mice (Fig 1A and S1A Fig). To assess *Smad4* deletion, we sorted tdTomato^+^ stromal cells from the bone marrow of *Osx-Cre*, *Ai9*, *Smad4*^*fl/fl*^ mice. Expression of full length *Smad4* mRNA was essentially absent, confirming efficient deletion of *Smad4* in bone marrow MSCs (Fig 1B).

Immunostaining of bone sections from *Osx-Cre*, *Ai9*, *Smad4*^*fl/fl*^ mice suggested that the number and organization of bone marrow MSCs was comparable to control mice (Fig 1A, and S1 Fig). To further characterize the hematopoietic niche, we quantified the number of PDGFRβ^+^ Sca1^+^ CD31^-^ lineage^-^ cells (mesenchymal stem/progenitor cells) and PDGFRβ^+^ Sca1^-^ CD31^-^ lineage^-^ cells (a mixture of CAR cells and osteoblasts) by flow cytometry (Fig 1C). We also quantified CD31^+^ Sca1^-^ lineage^-^ venous sinusoidal cells and CD31^+^ Sca1^+^ lineage^-^ arteriolar endothelial cells. In each case, the number of stromal cells was similar to control mice (Fig 1D). We previously reported that loss of TGF-β signaling in MSCs during development results in a marked increase in bone marrow adiposity. However, no increase in bone marrow adipocytes was observed in *Osx-Cre*, *Smad4*^*fl/fl*^ mice (S1B Fig). Finally, total bone marrow expression of key niche factors, including *Spp1*, *Scf*, and *Cxcl12*, was comparable in *Osx-Cre*, *Smad4*^*fl/fl*^ and control mice (Fig 1E). Together, these data suggest SMAD4 signaling in bone marrow MSCs is not required for their maintenance under steady-state conditions.

### Post-natal loss of *Smad4* in MSCs does not alter basal hematopoiesis

**Fig 2:**
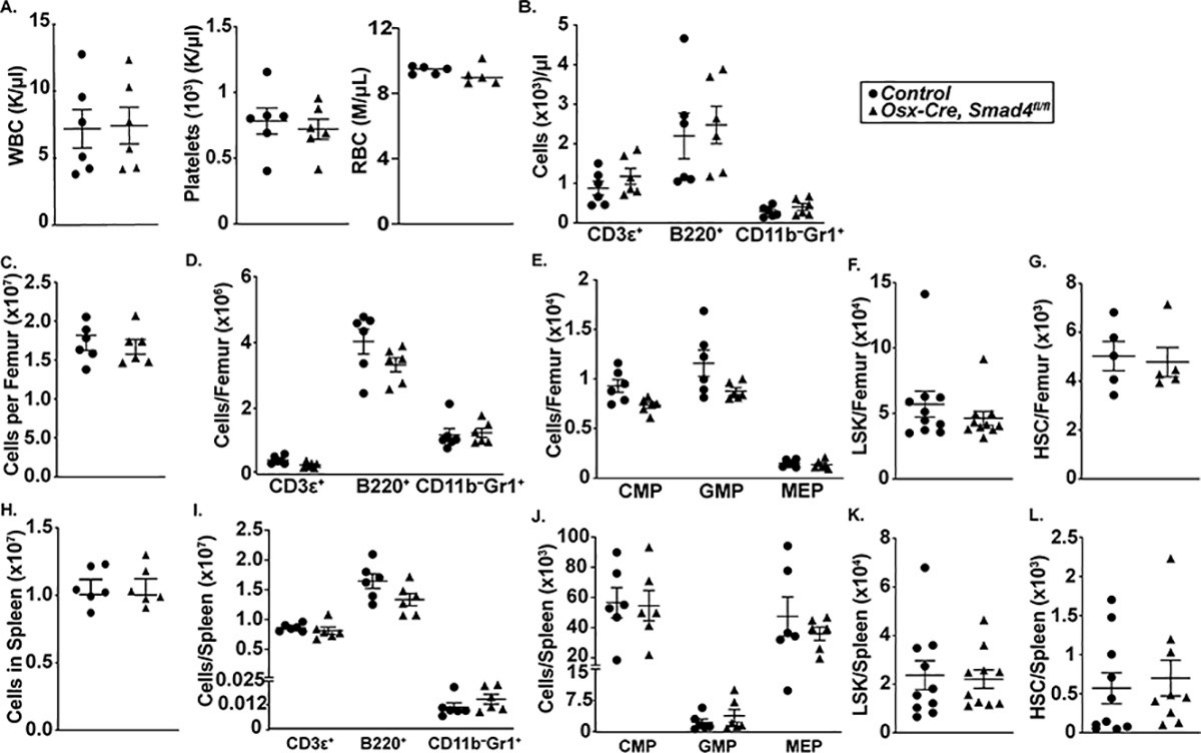
Basal hematopoiesis is normal in *Osx-Cre Smad4*^*fl/fl*^ mice. (A) Peripheral counts in *Osx-Cre* (control) or *Osx-Cre Smad4*^*fl/fl*^ mice. (B) Number of circulating CD3ε^+^ (T-lineage), B220^+^ (B-lineage), and CD11b^+^ Gr1^+^ (Granulocytes). (C) Bone marrow cellularity. (D) Number of CD3ε^+^, B220^+^, and CD11b^–^ Gr1^+^ cells per femur. (E) Number of common myeloid progenitors (CMP, lineage^−^Sca1^–^ Kit^+^ CD34^+^ cells), granulocyte-macrophage progenitors (GMP, lineage^−^Sca1^–^ Kit^+^ CD34^+^ CD16/32^+^ cells), and megakaryocyte-erythrocyte progenitors (MEP, lineage^−^Sca1^–^ Kit^+^ CD34^–^ CD16/32^–^ cells) per femur. (F) Number of phenotypic hematopoietic stem/progenitor cells (lineage^−^Sca1^+^ Kit^+^, LSK cells) per femur. (G) Number of phenotypic hematopoietic stem cells (LSK CD48^–^ CD41^–^ CD150^+^ cells) per femur. (H) Spleen cellularity. (I) Number of CD3ε^+^, B220^+^, and CD11b^+^ Gr1^+^ per spleen. (J) Number of CMP, GMP, and MEP per spleen. (K) Number of LSK cells per spleen. (L) Number of phenotypic HSCs per spleen. Data represent the mean ± SEM.

We next examined basal hematopoiesis in *Osx-Cre*, *Smad4*^*fl/fl*^ mice. Peripheral blood counts and the level of circulating T cells, B cells, and neutrophils were normal (Fig 2A and 2B). Likewise, the number of myeloid, B-cell, and T-cell lineage cells in the bone marrow was comparable to control mice (Fig 2C and 2D). The number of phenotypic hematopoietic stem cells (HSCs), common myeloid progenitors (CMPs), granulocyte-macrophage progenitors (GMPs), megakaryocyte-erythrocyte progenitors (MEPs), and LSK cells in the bone marrow of *Osx-Cre*, *Smad4*^*fl/fl*^ also was similar to control mice (Fig 2E–2G). Finally, no perturbation in the number of mature hematopoietic cells or HSPCs in the spleen of *Osx-Cre*, *Smad4*^*fl/fl*^ mice was observed (Fig 2H–2L). Collectively, these data show that SMAD4 signaling in bone marrow mesenchymal cells is not required to maintain basal hematopoiesis.

### Post-natal loss of *Smad4* in MSCs does not alter basal or stress erythropoiesis

**Fig 3:**
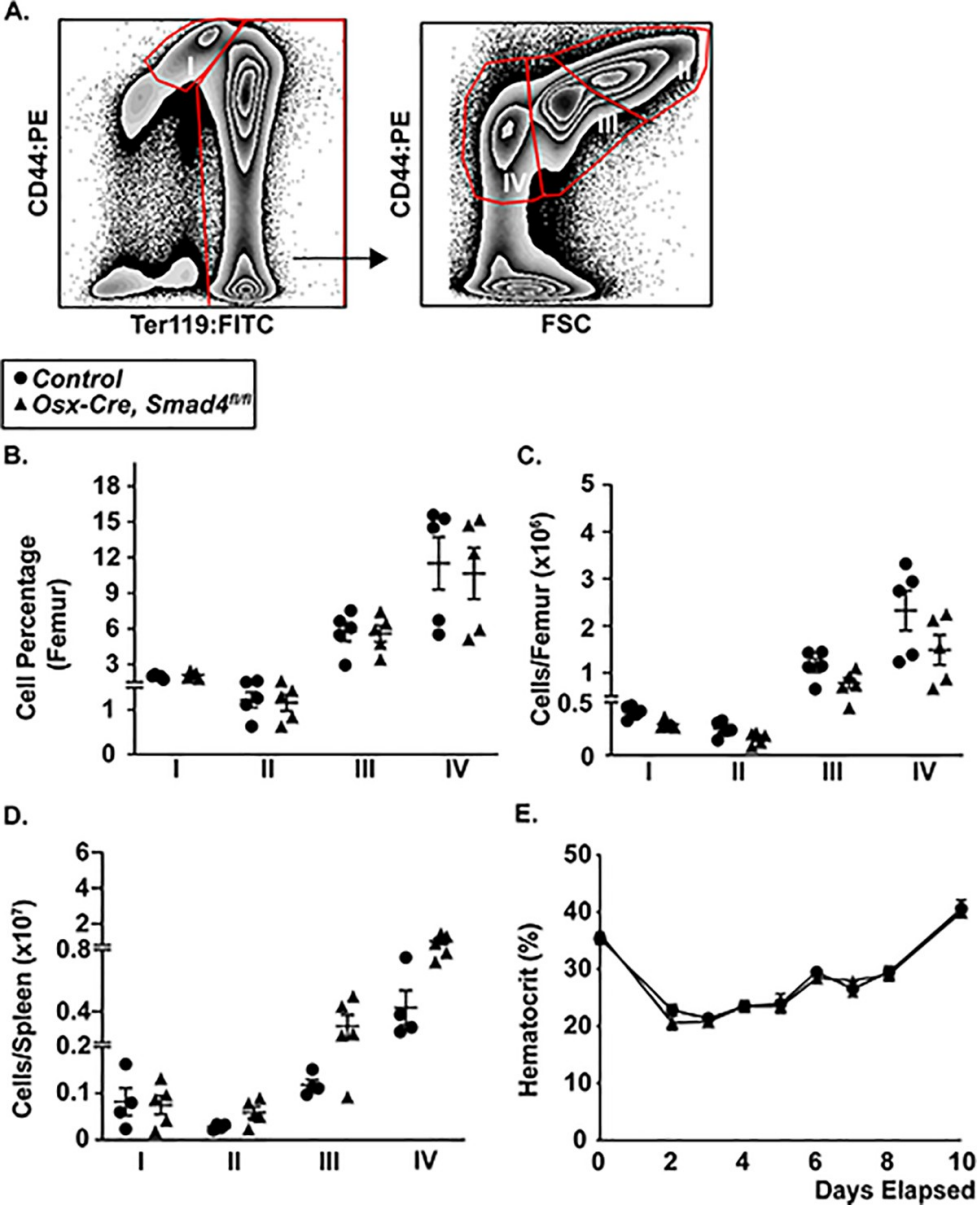
Erythropoiesis is normal in *Osx-Cre Smad4*^*fl/fl*^ mice. (A) Representative flow plots showing the gating strategy to identify different stages of erythroid development: stage I proerythroblasts (CD44^high^ Ter119^int^ cells); stage II basophilic erythroblasts (CD44^high^, Ter119^high^ forward scatter^high^ cells); stage III (polychromatic erythroblasts, (CD44^high^, Ter119^high^ forward scatter^int^ cells); and stage IV orthochromatic erythroblasts (CD44^high^, Ter119^high^ forward scatter^high^ cells). (B) Percentage of erythroid progenitors in the bone marrow. (C) Absolute number of erythroid progenitors per femur. (D) Absolute number of erythroid progenitors per spleen. (E) Mice were treated with a single 30 mg/kg dose of phenylhydrazine to induce hemolysis on day 0. Shown is the hematocrit at the indicated time after phenylhydrazine. Data represent the mean ± SEM. Significance determined by one-way ANOVA.

There is evidence that activin signaling in bone marrow stromal cells may contribute to the regulation of erythropoiesis.[23] Flow cytometry was used to identify and quantify phenotypic erythroid progenitors in bone marrow and spleen (Fig 3A). The number of stage I proerythroblasts (CD44^high^ Ter119^int^ cells), stage II basophilic erythroblasts (CD44^high^, Ter119^high^ forward scatter^high^ cells), stage III (polychromatic erythroblasts, (CD44^high^, Ter119^high^ forward scatter^int^ cells), and stage 4 orthochromatic erythroblasts (CD44^high^, Ter119^high^ forward scatter^high^ cells) in the bone marrow or spleen was similar in control and *Osx-Cre*, *Smad4*^*fl/fl*^ mice (Fig 3B–3D).

To assess stress erythropoiesis, we characterized the response of mice to the induction of acute hemolytic anemia after phenylhydrazine treatment. As expected, in control mice, treatment with phenylhydrazine induced an acute fall in the hematocrit followed by recovery over a 10-day period (Fig 3E). The magnitude of anemia and kinetics of recovery were similar in *Osx-Cre*, *Smad4*^*fl/fl*^ mice. These data show that SMAD4-dependent signaling in mesenchymal stromal cells is not required for the suppressive effect of activins on erythropoiesis.

### Post-natal loss of *Smad4* in mesenchymal stromal cells does not alter hematopoietic recovery following myeloablative chemotherapy

**Fig 4:**
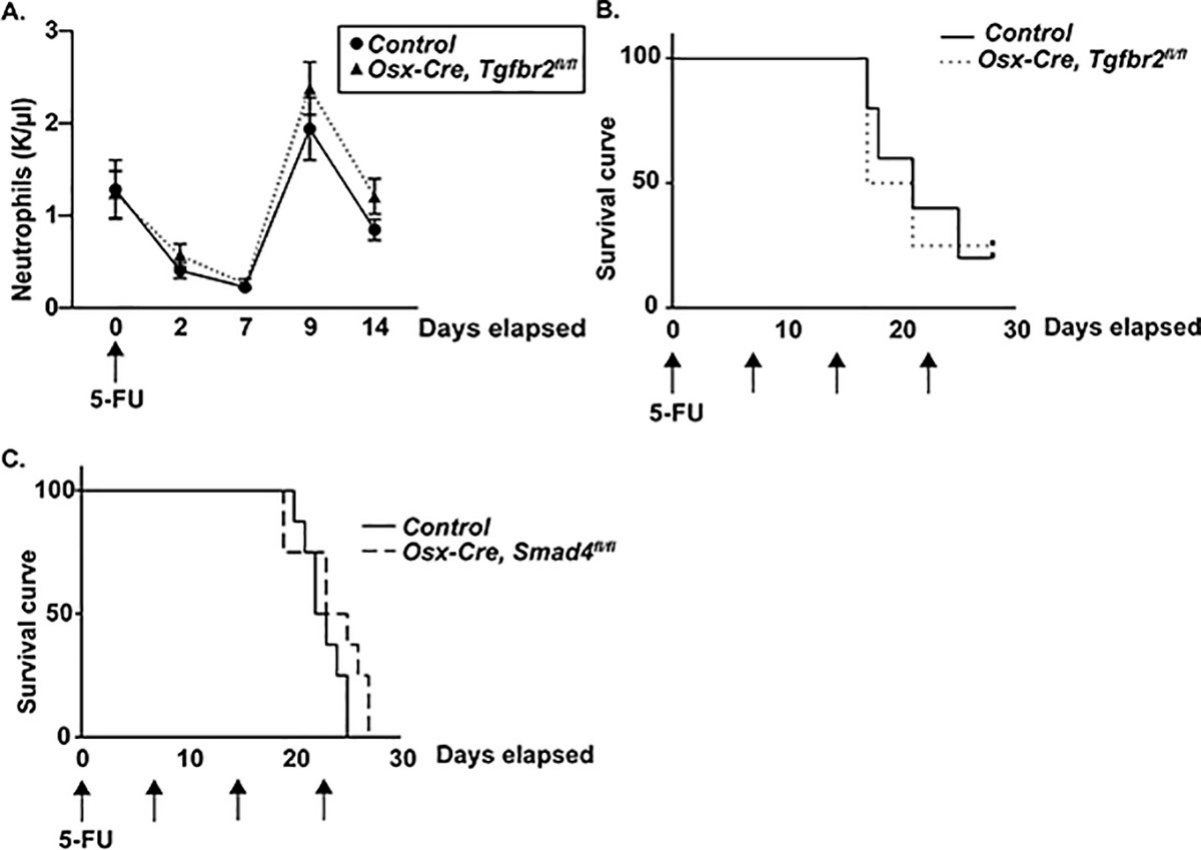
TGF-β signaling in mesenchymal stromal cells is not required for hematopoietic recovery following myeloablation with 5-FU. (A) *Osx-Cre Tgfbr2*^*fl/fl*^ or *Osx-Cre* (control) mice were treated with a single 150 mg/kg dose of 5-FU and hematopoietic recovery assessed by serial blood counts (n = 5 per cohort). (B) *Osx-Cre Tgfbr2*^*fl/fl*^ or control mice were treated weekly with 150 mg/kg of 5-FU for 4 weeks to induce HSC exhaustion. Shown is mouse survival (n = 5 per cohort). (C) *Osx-Cre Smad4*^*fl/fl*^ or control mice were treated weekly with 150 mg/kg of 5-fluorouracil (5-FU) for 4 weeks to induce HSC exhaustion. Shown is mouse survival (n = 8 per cohort). Data represent the mean ± SEM. Significance determined by Log-rank Mantel-Cox test with Gehan-Breslow-Wilcoxon test.

A prior study suggested that TGF-β contributes to hematopoietic recovery following myeloablative therapy. However, the contribution of TGF-β signaling in MSCs to hematopoietic recovery is unknown. To address this question, we characterized the hematopoietic response to 5-fluorouracil (5-FU) in *Osx-Cre*, *Tgfbr2* ^*fl/fl*^ mice, in which both canonical and non-canonical TGF-β signaling is abrogated in MSCs. Of note, we previously reported that basal hematopoiesis was normal in these mice when *Osx-Cre* was activated on day 1–2 after birth.[12] In control mice, treatment with a single dose of 5-FU induced neutropenia with a nadir on day 7 and complete recovery by day 9 (Fig 4A). Both the magnitude of neutropenia induced by 5-FU and kinetics of neutrophil recovery were similar in *Osx-Cre Tgfbr2*^*fl/fl*^ mice. Repeated doses of 5-FU induce HSC exhaustion and death in mice due to hematopoietic failure. Similar median survival after weekly 5-FU was observed in *Osx-Cre*, *Tgfbr2*^*fl/fl*^ and control mice (Fig 4B). These data show that TGF-β signaling in bone marrow MSCs does not contribute to hematopoietic recovery following myeloablative therapy. Of note, survival following repeated treatment with 5-FU in *Osx-Cre*, *Smad4*^*fl/fl*^ mice was comparable to control mice, suggesting that canonical signaling by other TGF-β family members in MSCs also does not contribute to hematopoietic stress responses to myeloablative therapy (Fig 4C).

### Loss of *Smad4* in mesenchymal stromal cells does not affect HSPC mobilization by granulocyte-colony stimulating factor

**Fig 5:**
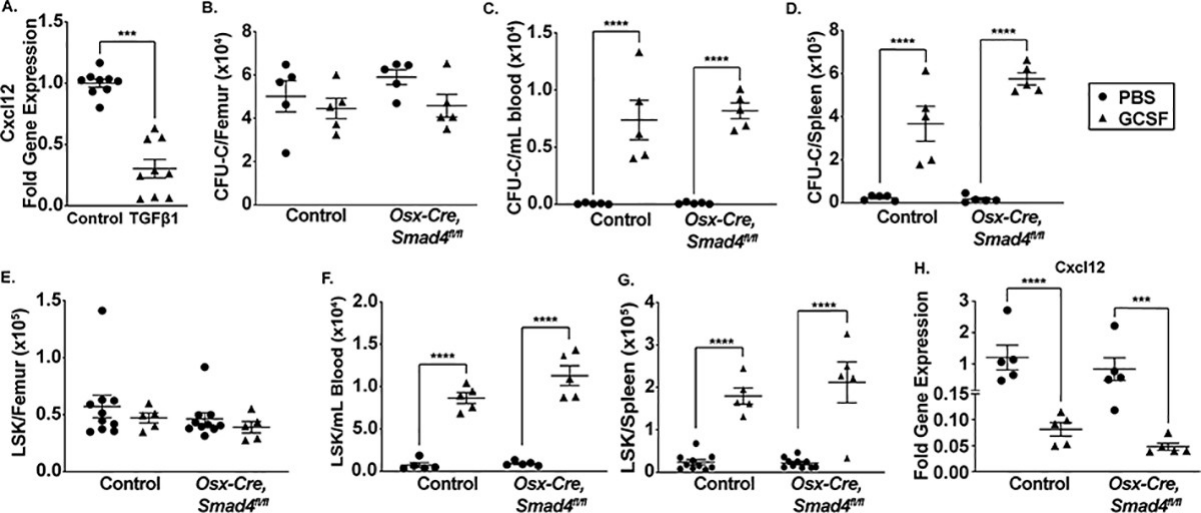
TGF-β signaling in mesenchymal stromal cells is not required for G-CSF induced HSPC mobilization. (A) Bone marrow mesenchymal stromal cell cultures were established from wildtype mice. Cells were treated with TGFβ1 ligand (10 ng/ml) for 72 hours and then RNA harvested. Shown is *Cxcl12* mRNA expression relative to *β-actin* mRNA. ***P < 0.001 by unpaired t-test. (B-D) *Osx-Cre Smad4*^*fl/fl*^ or control mice were treated with 125 mg/kg of granulocyte-colony stimulating factor (G-CSF) or saline alone twice daily for 7 days. Shown are the number of colony forming cells (CFU-C) in bone marrow (B), blood (C) or spleen (D). (E-F) Shown are the number of LSK cells in bone marrow (E), blood (F), or spleen (G) after 7 days of G-CSF or saline treatment. Data represent the mean ± SEM. Significance determined by two-way ANOVA with alpha of 0.05 and Sidak’s multiple comparisons test. ***P< 0.001 ****P < 0.0001.

There is strong evidence that G-CSF induced HSPC mobilization is mediated, at least in part, by the downregulation of CXCL12 expression in bone marrow MSCs.[34–39] Treatment of bone marrow stromal cell lines with TGF downregulates CXCL12 expression in a SMAD4-dependent fashion.[40,41] There also is evidence that G-CSF treatment is associated with increased circulating TGF-β1 levels.[42] Together, these observations suggest the hypothesis that canonical TGF-β family signaling in bone marrow MSCs, by downregulating CXCL12 expression, may contribute to G-CSF induced HSC mobilization. To test this hypothesis, we first asked whether TGF-β1 regulates CXCL12 in primary murine bone marrow MSCs. Indeed, treatment of cultures of primary bone marrow MSC with TGF-β1 for 3 days resulted in a significant decrease in CXCL12 mRNA expression (Fig 5A). We next characterized G-CSF induced HSPC mobilization in *Osx-Cre*, *Smad4*^*fl/fl*^ mice. Treatment of control mice with G-CSF for 5 days induced robust mobilization of colony-forming cells (CFU-C) and Lineage^-^ Sca^+^ Kit^+^ (LSK) cells into the blood and spleen (Fig 5B–5F). A similar level of HSPC mobilization was observed in *Osx-Cre*, *Smad4*^*fl/fl*^ mice. Consistent with prior studies, G-CSF induced a marked decrease in total bone marrow CXCL12 mRNA expression, with a similar decrease observed in *Osx-Cre*, *Smad4*^*fl/fl*^ mice (Fig 5G). We also analyzed G-CSF induced HSPC mobilization in *Osx-Cre*, *Tgfbr2*^*fl/fl*^ mice, where all TGF-β1 signaling in MSCs is abrogated. Again, HPSC mobilization by G-CSF, as measured by CFU-C levels in blood and spleen, was comparable to control mice (S2 Fig). Collectively, these data show that neither TGF-β1 signaling nor canonical TGF family signaling in bone marrow MSCs is required for efficient HSPC mobilization by G-CSF.

## Discussion

Recent single cell RNA sequencing studies of murine bone marrow stromal cells show that most of the receptors for TGF family members are expressed at low levels on mesenchymal stromal cells, including osteolineage cells, isolated from adult mice under steady state conditions. Prior studies have established that TGF family members play an important role during development in the generation of the hematopoietic niche in the bone marrow. In particular, we recently showed that TGF-β signaling plays a key role in the specification of fetal mesenchymal progenitor cells to a non-adipocyte fate. There also is evidence, mainly from cell culture systems, that TGF family members can regulate the function of mesenchymal stromal cells. In contrast, our data suggest that canonical SMAD4-dependent TGF family member signaling in mesenchymal stromal cells is dispensable for their maintenance in the bone marrow hematopoietic niche, once the niche is established. Of note, since *Osx-Cre* only targets mesenchymal stromal cells in the bone marrow, the contribution of TGF family member signaling in other stromal cells, such as endothelial cells, to basal and stress hematopoiesis remains uncertain.

There is evidence that alterations in mesenchymal stromal cells contribute to stress hematopoiesis responses. G-CSF induced HSPC mobilization is associated with a loss of active osteoblasts and decreased CXCL12 expression in bone marrow MSCs. Myeloablation due to chemotherapy or radiation therapy is associated with an expansion of bone marrow adipocytes, which support hematopoietic recovery through secretion of stem cell factor.[29] Myeloablative radiation also induces a loss of osteoblasts, which is followed by an expansion of osteolineage cells.[28] Our data suggest that SMAD-dependent signaling by TGF family members in bone marrow MSCs is not a major contributor to hematopoiesis responses to certain stressors, including myeloablation therapy with 5-FU, acute anemia, and G-CSF treatment. Whether TGF family member signaling contributes to hematopoiesis responses after other stressors, such as irradiation needs further study.

TGF family members activate multiple intracellular signaling pathways besides SMADs, including Erk[43–45], Rho-like GTPases[46–51], JNK/p38[52,53], and PI3K/Akt[54–56]. Our prior and current data suggest that both SMAD-dependent and -independent TGF-β signaling in bone marrow MSCs is dispensable for hematopoietic niche maintenance and response to certain stressors. However, for the other TGF family members, there is a possibility that non-SMAD dependent signaling may regulate bone marrow MSCs. Indeed, a prior study showed that loss of BMP receptor type 1A (*Bmpr1a*) in bone marrow MSCs results in an expansion of N-cadherin^+^ osteoblastic cells and an increase in HSCs.[21] Our data suggests that non-SMAD4 dependent BMP signaling likely mediates this response. Further studies are needed to test this hypothesis and characterize the contribution of non-canonical signaling by TGF family members in bone marrow MSCs to the regulation of basal or stress hematopoiesis.

## Materials and methods

### Contact for reagents and resource sharing

Further information and requests for resources and reagents should be directed to Daniel C. Link (danielclink@wustl.edu).

### Mice and animal housing

*Osx1-GFP*::*Cre*[57], *Ai9*[58], *Smad4* ^fl/fl^ [59], and *Tgfbr2*^*fl/fl*^ [60] mice were obtained from The Jackson Laboratory (Bar Harbor, ME). Mice were crossed to generate *Osx-Cre Tgfbr2*^*fl/fl*^, *Osx-Cre Ai9 Tgfbr2*^*fl/fl*^, *Osx-Cre Smad4*^*fl/fl*^, and *Osx-Cre Ai9 Smad4*^*fl/fl*^ mice on a C57Bl/6 background. To suppress the *Osx-Cre* transgene throughout embryonic development, mice were maintained on doxycycline chow (200 mg/serving) until post-natal day 0. All experiments were done using 6-8-week-old mice. An equal number of male and female mice were used. Mice were maintained under standard pathogen-free conditions, and all of the procedures performed in this study were approved by the Washington University Animal Studies Committee (approval number 20180018). Mice were anesthetized using isoflurane for all procedures, and they were euthanized using CO_2_ asphyxiation followed by cervical dislocation.

### Flow cytometry

Peripheral blood, bone marrow, and spleen mononuclear cell (MNC) preparations were suspended in Tris-buffered ammonium chloride (pH 7.2) buffer for 15 minutes at room temperature (RT) to lyse red blood cells. MNCs were then incubated with target antibodies at 4°C for 30 minutes in phosphate buffered saline (PBS) containing 1mM ethylenediaminetetraacetic acid (EDTA) and 0.2% (weight/volume) bovine serum albumin (BSA). The HSPC panel included Pe-Cy7-conjugated CD117 (2B8); BV711-conjugated Ly-6A/E; BV605-conjugated CD150 (TC15-12F12.2) BV421-conjugated CD48 (HM48-1); APC-conjugated CD16/32 (2.4G2); FITC-conjugated CD34 (RAM34); and PE-conjugated CD135 (A2F10.1) and the following APC-Cy7-conjugated antibodies recognizing lineage markers: CD3e (145-2C11), B220 (RA3-6B2), Gr1 (RB6-8C5), Ter119 (TER-119), and CD11b (M1/70). The mesenchymal cell panel included PerCP-Cy5.5-conjugated Ly-6A/E (D7), BV421-conjugated CD31 (390), biotin-conjugated PDGFRβ (APB5), PE-conjugated streptavidin, APC-Cy7-conjugated CD45 (30-F11) and Ter119 (TER-119) antibodies. The lineage cell panel included PE-conjugated CD115 (CSF-1R), FITC-conjugated Ly-6G/Ly-6C (RB6-8C5), BV421-conjugated CD45R/B220 (RA3-6B2), and APC-Cy7-conjugated CD3ε (145-2C11). The erythroid panel included FITC-conjugated Ter119 (TER-119), PE-conjugated CD44 (IM7) and the following APC-Cy7-conjugated antibodies: Ly-6G/Ly-6C (RB6-8C5), CD45R/B220 (RA3-6B2), and CD3ε (145-2C11). All antibodies were obtained from BioLegend (San Diego, CA), unless otherwise indicated. Data were acquired using a FACS Aria III flow cytometer (BD biosciences, San Jose, CA) and analyzed using FlowJo^TM^ v10.6.1 software (BD biosciences).

### Cell sorting

Mouse femurs, tibias, and pelvises were homogenized in PBS using a mortar and pestle. The cell suspension was then incubated in PBS containing 1.7 mg/ml collagenase type 1 (#17100017, ThermoFisher), 1.7 mg/ml collagenase type 2 (#LS004174, Worthington, Lakewood, NJ), and 1.7 mg/ml collagenase type 4 (#LS004188, Worthington) at 37°C while shaking for 15 minutes. The resulting cell suspension was filtered through CellTrics 50μm filters (Sysmex, Goerlitz, Germany) to remove debris, creating a single-cell suspension. The samples were then incubated at 4°C for 30 minutes in PBS containing 1mM EDTA and 0.2% BSA with the following panel of BV421-conjugated lineage antibodies: CD45, Gr1, CD11b, and B220. Ai9^+^ lineage^-^ cells were sorted using a Sony iCyt Synergy SY3200 (Synergy)” cell sorter (Sony, San Jose, CA).

*Immunostaining of bone sections*. Mouse femurs were fixed in PBS containing 4% paraformaldehyde, pH 7.4, for 24 hours at 4°C. Bones were then decalcified in PBS containing 14% EDTA, pH 7.4, for 14 days at 4°C, changing buffer every 24 hours. Following incubation in PBS containing 30% sucrose for 1 hour at room temperature, bones were embedded in optimal cutting temperature compound (OCT) (Sakura Finetek, Torrance, CA). The tissue blocks were cut into 12μm sections using a Leica Cryo-Jane system (Leica Biosystems, Wetzlar, Germany). For immunostaining, the slides were first incubated in 0.1M Tris-Cl pH 7.5, 150mM NaCl, and 0.1% Tween 20 (TNT) buffer containing 10% donkey serum for 1 hour at room temperature. Sections were then incubated for 15 minutes at room temperature using the Avidin/Biotin Blocking Kit (SP-2001; Vector Laboratories, Burlingame, CA). Sections were incubated with the primary antibody overnight at 4°C and, where applicable, then incubated with the secondary antibody at a 1 to 100 dilution for 1 hour at room temperature. The following antibodies were used: rabbit anti-osteocalcin (FL-95) at a 1:50 dilution (Santa Cruz Biotechnology Dallas, TX) and goat anti-VE-cadherin (AF1002) at a 1:25 dilution (R&D Systems Minneapolis, MN). Finally, slides were mounted with ProLong Gold antifade reagent with DAPI (Life Technologies, Inc., Grand Island, NY). Images were acquired using an LSM 700 confocal microscope (Carl Zeiss Microscopy, Peabody, MA) and processed using Volocity® v6.5.1 software (PerkinElmer, Waltham, MA). Oil red staining was performed using the Sigma Oil Red O kit per manufacturer’s recommendations (Millipore Sigma Darmstadt, Germany). Sections were mounted with Organo/Limonene Mount™ (Millipore Sigma), and images were acquired using a Hamamatsu Nanozoomer (Hamamatsu Photonics, Hamamatsu City, Japan).

### Quantitative reverse-transcription PCR

Total bone marrow RNA was obtained by flushing femurs with 1 ml of Trizol (Invitrogen). RNA was prepared according to the manufacturer’s specification. One-step quantitative reverse-transcription PCR was performed using 50 ng of total RNA and the iTaq^TM^ Universal Probes One-Step Kit (Bio Rad, Hercules, CA) with no template and no reverse transcriptase controls. mRNA expression is normalized to *β-actin* mRNA expression. Data was collected on a StepOnePlus^TM^ Real-Time PCR System (Thermo Fisher). The following quantitative PCR primer/probe sets were used:

TaqMan® Assays and Arrays:

Actb, Mus_musculus VIC–spanning exons 1–2 Mm04394036_g1
Kit ligand, Mus_musculus FAM–spanning exons 2–3 Mm00442972_m1
Cxcl12, Mus_musculus FAM–spanning exons 2–3 Mm00445553_m1
Spp1, Mus_musculus FAM–spanning exons 3–4 Mm00436767_m1
IDT PrimeTime Std® qPCR Assay:
Smad4, Mus_musculus FAM–spanning exons 8–9 Mm.PT.58.31543505

### Mesenchymal stromal cell culture

C57BL/6 mice were sacrificed at 3-to-4-weeks-old, and hindlimb bones were harvested and mechanically disrupted in complete Dulbecco’s modified eagle medium (DMEM) containing 20% fetal bovine serum (FBS) and penicillin-streptomycin using a mortar and pestle. The resulting cell suspension was cultured overnight at 37°C with 5% CO_2_, and the following day non-adherent cells removed by gentle aspiration. Adherent cells were cultured until reaching approximately 40% confluence (generally, 5–6 days). 100,000–200,000 cells were plated in 6 well plates 24 hours prior to treatment. TGFβ1 (14-8342-80, Life Technologies) was added every 24 hours for 72 hours at a dose of 10ng/mL.

### Colony-forming unit assay

25μL of peripheral blood, or 50,000 bone marrow MNCs, or 50,000 spleen MNCs were suspended in DMEM containing 2% FBS and 1% penicillin-streptomycin. The cell suspensions were mixed with 2 mL of methocult (M3434, Stemcell Technologies, Vancouver, Canada) prior to plating 1.1 mL of the methocult-cell mix in one 35 mm dish, repeating with the remaining mix for two technical replicates. The dishes were placed in a 100 mm petri dish with a third 35 mm dish containing 3 mL of sterile water. The samples were incubated at 37°C, 5% CO_2_ in air and ≥ 95% humidity for 7–10 days prior to colony counting.

### Phenylhydrazine, 5-FU, and G-CSF treatments

Phenylhydrazine (30 mg/kg, Sigma) was given daily for 2 days by intraperitoneal injection. A single injection of 5-FU (150 mg/kg, F6627-5G, Sigma) was given by intraperitoneal injection. For the HSC exhaustion experiments, mice were treated with weekly 150mg/kg doses of 5-FU. Human G-CSF (125 mg/kg, Amgen, Thousand Oaks, CA) was given twice daily for 7 days via intraperitoneal injection. Complete blood counts were obtained using the HV950 hemavet (Drew Scientific, Miami Lakes, FL).

### Quantification and statistical analysis

Significance was determined using Prism v8.1.2 (GraphPad, San Diego, CA, USA). For single parameter analysis, unpaired t-test were used to assess statistical significance. For multiple parameter data, statistical significance was calculated using one-way or two-way analysis of variance (ANOVA). P values less than 0.05 were considered significant. Expression data are log transformed prior to statistical analysis.

## Supporting information

S1 Fig Characterization of mesenchymal stromal cells in *Osx-Cre*, *Ai9*, *Smad4*^*fl/fl*^ mice. (A) Representative photomicrographs of femur sections from *Osx-Cre Ai9* (control) or *Osx-Cre*, *Ai9*, *Smad4*^*fl/fl*^ (*Smad4*^*fl/fl*^) mice showing DAPI stained nuclei and tdTomato (Ai9) mesenchymal stromal cells with morphologic similarities to CAR cells. (B) Representative images of femurs stained with oil red o (purple/red staining) to identify adipocytes (black arrowheads). Femur sections from *Osx-Cre Tgfbr2*^*fl/fl*^ mice are included as a positive control, since we previously showed that constitutive deletion of *Tgfbr2* in mesenchymal stromal cells is associated with a marked increase in bone marrow adiposity (Abou-Ezzi, Stem Cell Reports, 2019).
(TIF)

S2 Fig G-CSF induced HSPC mobilization is normal in *Osx-Cre Tgfbr2*^*fl/fl*^ mice. (A-C) *Osx-Cre Tgfbr2*^*fl/fl*^ or *Osx-Cre* (control mice) were treated with 125 mg/kg of granulocyte-colony stimulating factor (G-CSF) twice daily for 7 days (). Shown are the number of colony forming cells (CFU-C) in bone marrow (A), blood (B) or spleen (C). D-E) Shown are the number of LSK cells in bone marrow (D) and spleen (E) after 7 days of G-CSF. Data represent the mean ± SEM. ***P < 0.001 and ****P < 0.0001 by two-way ANOVA with an alpha of 0.05 and Sidak’s multiple comparisons test. The saline treated cohort is the same as in Fig 5.
(TIF)

## References

[1] Ding L, Morrison SJ. Haematopoietic stem cells and early lymphoid progenitors occupy distinct bone marrow niches. *Nature*. 2013;495(7440):231–235. 23434755 10.1038/nature11885 PMC3600153

[2] Greenbaum A, Hsu YM, Day RB, et al CXCL12 in early mesenchymal progenitors is required for haematopoietic stem-cell maintenance. *Nature*. 2013;495(7440):227–230. 23434756 10.1038/nature11926 PMC3600148

[3] Kunisaki Y, Bruns I, Scheiermann C, et al Arteriolar niches maintain haematopoietic stem cell quiescence. *Nature*. 2013;502(7473):637–643. 24107994 10.1038/nature12612 PMC3821873

[4] Omatsu Y, Sugiyama T, Kohara H, et al The essential functions of adipo-osteogenic progenitors as the hematopoietic stem and progenitor cell niche. *Immunity*. 2010;33(3):387–399. 20850355 10.1016/j.immuni.2010.08.017

[5] Tokoyoda K, Egawa T, Sugiyama T, Choi BI, Nagasawa T. Cellular niches controlling B lymphocyte behavior within bone marrow during development. *Immunity*. 2004;20(6):707–718. 15189736 10.1016/j.immuni.2004.05.001

[6] Yu VW, Saez B, Cook C, et al Specific bone cells produce DLL4 to generate thymus-seeding progenitors from bone marrow. *J Exp Med*. 2015;212(5):759–774. 25918341 10.1084/jem.20141843 PMC4419348

[7] Zhu J, Garrett R, Jung Y, et al Osteoblasts support B-lymphocyte commitment and differentiation from hematopoietic stem cells. *Blood*. 2007;109(9):3706–3712. 17227831 10.1182/blood-2006-08-041384

[8] Chen G, Deng C, Li YP. TGF-beta and BMP signaling in osteoblast differentiation and bone formation. *Int J Biol Sci*. 2012;8(2):272–288. 22298955 10.7150/ijbs.2929 PMC3269610

[9] Alliston T, Choy L, Ducy P, Karsenty G, Derynck R. TGF-beta-induced repression of CBFA1 by Smad3 decreases cbfa1 and osteocalcin expression and inhibits osteoblast differentiation. *EMBO J*. 2001;20(9):2254–2272. 11331591 10.1093/emboj/20.9.2254 PMC125448

[10] Ignotz RA, Massague J. Type beta transforming growth factor controls the adipogenic differentiation of 3T3 fibroblasts. *Proc Natl Acad Sci U S A*. 1985;82(24):8530–8534. 3001708 10.1073/pnas.82.24.8530 PMC390950

[11] Sparks RL, Allen BJ, Strauss EE. TGF-beta blocks early but not late differentiation-specific gene expression and morphologic differentiation of 3T3 T proadipocytes. *J Cell Physiol*. 1992;150(3):568–577. 1537885 10.1002/jcp.1041500318

[12] Abou-Ezzi G, Supakorndej T, Zhang J, et al TGF-beta Signaling Plays an Essential Role in the Lineage Specification of Mesenchymal Stem/Progenitor Cells in Fetal Bone Marrow. *Stem Cell Reports*. 2019. 10.1016/j.stemcr.2019.05.017 PMC6626889 31204302

[13] Peters SB, Wang Y, Serra R. Tgfbr2 is required in osterix expressing cells for postnatal skeletal development. *Bone*. 2017;97:54–64. 28043895 10.1016/j.bone.2016.12.017 PMC5368008

[14] Herpin A, Lelong C, Favrel P. Transforming growth factor-beta-related proteins: an ancestral and widespread superfamily of cytokines in metazoans. *Dev Comp Immunol*. 2004;28(5):461–485. 15062644 10.1016/j.dci.2003.09.007

[15] Burt DW. Evolutionary grouping of the transforming growth factor-beta superfamily. *Biochem Biophys Res Commun*. 1992;184(2):590–595. 1575734 10.1016/0006-291x(92)90630-4

[16] Winnier G, Blessing M, Labosky PA, Hogan BL. Bone morphogenetic protein-4 is required for mesoderm formation and patterning in the mouse. *Genes Dev*. 1995;9(17):2105–2116. 7657163 10.1101/gad.9.17.2105

[17] Wozney JM, Rosen V, Celeste AJ, et al Novel regulators of bone formation: molecular clones and activities. *Science*. 1988;242(4885):1528–1534. 3201241 10.1126/science.3201241

[18] Dudley AT, Lyons KM, Robertson EJ. A requirement for bone morphogenetic protein-7 during development of the mammalian kidney and eye. *Genes Dev*. 1995;9(22):2795–2807. 7590254 10.1101/gad.9.22.2795

[19] Luo G, Hofmann C, Bronckers AL, Sohocki M, Bradley A, Karsenty G. BMP-7 is an inducer of nephrogenesis, and is also required for eye development and skeletal patterning. *Genes Dev*. 1995;9(22):2808–2820. 7590255 10.1101/gad.9.22.2808

[20] Mishina Y, Suzuki A, Ueno N, Behringer RR. Bmpr encodes a type I bone morphogenetic protein receptor that is essential for gastrulation during mouse embryogenesis. *Genes Dev*. 1995;9(24):3027–3037. 8543149 10.1101/gad.9.24.3027

[21] Zhang J, Niu C, Ye L, et al Identification of the haematopoietic stem cell niche and control of the niche size. *Nature*. 2003;425(6960):836–841. 14574412 10.1038/nature02041

[22] Suragani RN, Cadena SM, Cawley SM, et al Transforming growth factor-beta superfamily ligand trap ACE-536 corrects anemia by promoting late-stage erythropoiesis. *Nat Med*. 2014;20(4):408–414. 24658078 10.1038/nm.3512

[23] Iancu-Rubin C, Mosoyan G, Wang J, Kraus T, Sung V, Hoffman R. Stromal cell-mediated inhibition of erythropoiesis can be attenuated by Sotatercept (ACE-011), an activin receptor type II ligand trap. *Exp Hematol*. 2013;41(2):155–166 e117. 23261964 10.1016/j.exphem.2012.12.002

[24] Dussiot M, Maciel TT, Fricot A, et al An activin receptor IIA ligand trap corrects ineffective erythropoiesis in beta-thalassemia. *Nat Med*. 2014;20(4):398–407. 24658077 10.1038/nm.3468 PMC7730561

[25] Suragani RN, Cawley SM, Li R, et al Modified activin receptor IIB ligand trap mitigates ineffective erythropoiesis and disease complications in murine beta-thalassemia. *Blood*. 2014;123(25):3864–3872. 24795345 10.1182/blood-2013-06-511238 PMC4064330

[26] Abdulkadyrov KM, Salogub GN, Khuazheva NK, et al Sotatercept in patients with osteolytic lesions of multiple myeloma. *Br J Haematol*. 2014;165(6):814–823. 24650009 10.1111/bjh.12835 PMC4312883

[27] Carrancio S, Markovics J, Wong P, et al An activin receptor IIA ligand trap promotes erythropoiesis resulting in a rapid induction of red blood cells and haemoglobin. *Br J Haematol*. 2014;165(6):870–882. 24635723 10.1111/bjh.12838 PMC4282119

[28] Dominici M, Rasini V, Bussolari R, et al Restoration and reversible expansion of the osteoblastic hematopoietic stem cell niche after marrow radioablation. *Blood*. 2009;114(11):2333–2343. 19433859 10.1182/blood-2008-10-183459 PMC2745851

[29] Zhou BO, Yu H, Yue R, et al Bone marrow adipocytes promote the regeneration of stem cells and haematopoiesis by secreting SCF. *Nat Cell Biol*. 2017;19(8):891–903. 28714970 10.1038/ncb3570 PMC5536858

[30] Brenet F, Kermani P, Spektor R, Rafii S, Scandura JM. TGFbeta restores hematopoietic homeostasis after myelosuppressive chemotherapy. *J Exp Med*. 2013;210(3):623–639. 23440043 10.1084/jem.20121610 PMC3600905

[31] Tikhonova AN, Dolgalev I, Hu H, et al Author Correction: The bone marrow microenvironment at single-cell resolution. *Nature*. 2019;572(7767):E6. 31296938 10.1038/s41586-019-1394-x

[32] Maes C, Kobayashi T, Selig MK, et al Osteoblast precursors, but not mature osteoblasts, move into developing and fractured bones along with invading blood vessels. *Dev Cell*. 2010;19(2):329–344. 20708594 10.1016/j.devcel.2010.07.010 PMC3540406

[33] Sugiyama T, Kohara H, Noda M, Nagasawa T. Maintenance of the hematopoietic stem cell pool by CXCL12-CXCR4 chemokine signaling in bone marrow stromal cell niches. *Immunity*. 2006;25(6):977–988. 17174120 10.1016/j.immuni.2006.10.016

[34] Katayama Y, Battista M, Kao WM, et al Signals from the sympathetic nervous system regulate hematopoietic stem cell egress from bone marrow. *Cell*. 2006;124(2):407–421. 16439213 10.1016/j.cell.2005.10.041

[35] Christopher MJ, Link DC. Granulocyte colony-stimulating factor induces osteoblast apoptosis and inhibits osteoblast differentiation. *J Bone Miner Res*. 2008;23(11):1765–1774. 18597629 10.1359/JBMR.080612 PMC2685485

[36] Christopher MJ, Liu F, Hilton MJ, Long F, Link DC. Suppression of CXCL12 production by bone marrow osteoblasts is a common and critical pathway for cytokine-induced mobilization. *Blood*. 2009;114(7):1331–1339. 19141863 10.1182/blood-2008-10-184754 PMC2727413

[37] Levesque JP, Hendy J, Takamatsu Y, Simmons PJ, Bendall LJ. Disruption of the CXCR4/CXCL12 chemotactic interaction during hematopoietic stem cell mobilization induced by GCSF or cyclophosphamide. *J Clin Invest*. 2003;111(2):187–196. 12531874 10.1172/JCI15994 PMC151860

[38] Petit I, Szyper-Kravitz M, Nagler A, et al G-CSF induces stem cell mobilization by decreasing bone marrow SDF-1 and up-regulating CXCR4. *Nat Immunol*. 2002;3(7):687–694. 12068293 10.1038/ni813

[39] Semerad CL, Christopher MJ, Liu F, et al G-CSF potently inhibits osteoblast activity and CXCL12 mRNA expression in the bone marrow. *Blood*. 2005;106(9):3020–3027. 16037394 10.1182/blood-2004-01-0272 PMC1895331

[40] Wright N, de Lera TL, Garcia-Moruja C, et al Transforming growth factor-beta1 down-regulates expression of chemokine stromal cell-derived factor-1: functional consequences in cell migration and adhesion. *Blood*. 2003;102(6):1978–1984. 12775566 10.1182/blood-2002-10-3190

[41] Khurana S, Melacarne A, Yadak R, et al SMAD signaling regulates CXCL12 expression in the bone marrow niche, affecting homing and mobilization of hematopoietic progenitors. *Stem Cells*. 2014;32(11):3012–3022. 25069965 10.1002/stem.1794 PMC4220608

[42] Kaygusuz MA, Turan CC, Aydin NE, et al The effects of G-CSF and naproxen sodium on the serum TGF-beta1 level and fracture healing in rat tibias. *Life Sci*. 2006;80(1):67–73. 17023006 10.1016/j.lfs.2006.08.023

[43] Mulder KM, Morris SL. Activation of p21ras by transforming growth factor beta in epithelial cells. *J Biol Chem*. 1992;267(8):5029–5031. 1544886

[44] Hartsough MT, Mulder KM. Transforming growth factor beta activation of p44mapk in proliferating cultures of epithelial cells. *J Biol Chem*. 1995;270(13):7117–7124. 7706248 10.1074/jbc.270.13.7117

[45] Galliher AJ, Schiemann WP. Src phosphorylates Tyr284 in TGF-beta type II receptor and regulates TGF-beta stimulation of p38 MAPK during breast cancer cell proliferation and invasion. *Cancer Res*. 2007;67(8):3752–3758. 17440088 10.1158/0008-5472.CAN-06-3851

[46] Frey RS, Mulder KM. Involvement of extracellular signal-regulated kinase 2 and stress-activated protein kinase/Jun N-terminal kinase activation by transforming growth factor beta in the negative growth control of breast cancer cells. *Cancer Res*. 1997;57(4):628–633. 9044838

[47] Engel ME, McDonnell MA, Law BK, Moses HL. Interdependent SMAD and JNK signaling in transforming growth factor-beta-mediated transcription. *J Biol Chem*. 1999;274(52):37413–37420. 10601313 10.1074/jbc.274.52.37413

[48] Hocevar BA, Brown TL, Howe PH. TGF-beta induces fibronectin synthesis through a c-Jun N-terminal kinase-dependent, Smad4-independent pathway. *EMBO J*. 1999;18(5):1345–1356. 10064600 10.1093/emboj/18.5.1345 PMC1171224

[49] Hanafusa H, Ninomiya-Tsuji J, Masuyama N, et al Involvement of the p38 mitogen-activated protein kinase pathway in transforming growth factor-beta-induced gene expression. *J Biol Chem*. 1999;274(38):27161–27167. 10480932 10.1074/jbc.274.38.27161

[50] Bhowmick NA, Zent R, Ghiassi M, McDonnell M, Moses HL. Integrin beta 1 signaling is necessary for transforming growth factor-beta activation of p38MAPK and epithelial plasticity. *J Biol Chem*. 2001;276(50):46707–46713. 11590169 10.1074/jbc.M106176200

[51] Yu L, Hébert MC, Zhang YE. TGF-beta receptor-activated p38 MAP kinase mediates Smad-independent TGF-beta responses. *EMBO J*. 2002;21(14):3749–3759. 12110587 10.1093/emboj/cdf366 PMC126112

[52] Bhowmick NA, Ghiassi M, Bakin A, et al Transforming growth factor-beta1 mediates epithelial to mesenchymal transdifferentiation through a RhoA-dependent mechanism. *Mol Biol Cell*. 2001;12(1):27–36. 11160820 10.1091/mbc.12.1.27 PMC30565

[53] Edlund S, Landström M, Heldin CH, Aspenström P. Transforming growth factor-beta-induced mobilization of actin cytoskeleton requires signaling by small GTPases Cdc42 and RhoA. *Mol Biol Cell*. 2002;13(3):902–914. 11907271 10.1091/mbc.01-08-0398 PMC99608

[54] Bakin AV, Tomlinson AK, Bhowmick NA, Moses HL, Arteaga CL. Phosphatidylinositol 3-kinase function is required for transforming growth factor beta-mediated epithelial to mesenchymal transition and cell migration. *J Biol Chem*. 2000;275(47):36803–36810. 10969078 10.1074/jbc.M005912200

[55] Shin I, Bakin AV, Rodeck U, Brunet A, Arteaga CL. Transforming growth factor beta enhances epithelial cell survival via Akt-dependent regulation of FKHRL1. *Mol Biol Cell*. 2001;12(11):3328–3339. 11694570 10.1091/mbc.12.11.3328 PMC60258

[56] Wilkes MC, Mitchell H, Penheiter SG, et al Transforming growth factor-beta activation of phosphatidylinositol 3-kinase is independent of Smad2 and Smad3 and regulates fibroblast responses via p21-activated kinase-2. *Cancer Res*. 2005;65(22):10431–10440. 16288034 10.1158/0008-5472.CAN-05-1522

[57] Rodda SJ, McMahon AP. Distinct roles for Hedgehog and canonical Wnt signaling in specification, differentiation and maintenance of osteoblast progenitors. *Development*. 2006;133(16):3231–3244. 16854976 10.1242/dev.02480

[58] Madisen L, Zwingman TA, Sunkin SM, et al A robust and high-throughput Cre reporting and characterization system for the whole mouse brain. *Nat Neurosci*. 2010;13(1):133–140. 20023653 10.1038/nn.2467 PMC2840225

[59] Yang X, Li C, Herrera PL, Deng CX. Generation of Smad4/Dpc4 conditional knockout mice. *Genesis*. 2002;32(2):80–81. 11857783 10.1002/gene.10029

[60] Leveen P, Larsson J, Ehinger M, et al Induced disruption of the transforming growth factor beta type II receptor gene in mice causes a lethal inflammatory disorder that is transplantable. *Blood*. 2002;100(2):560–568. 12091349 10.1182/blood.v100.2.560

