# Supplementary figures and images for: Canonical signaling by TGF family members in mesenchymal stromal cells is dispensable for hematopoietic niche maintenance under basal and stress conditions
Source: PLoS One. 2020 May 29;15(5):e0233751. doi: 10.1371/journal.pone.0233751 (PMC7259882; doi:10.1371/journal.pone.0233751)

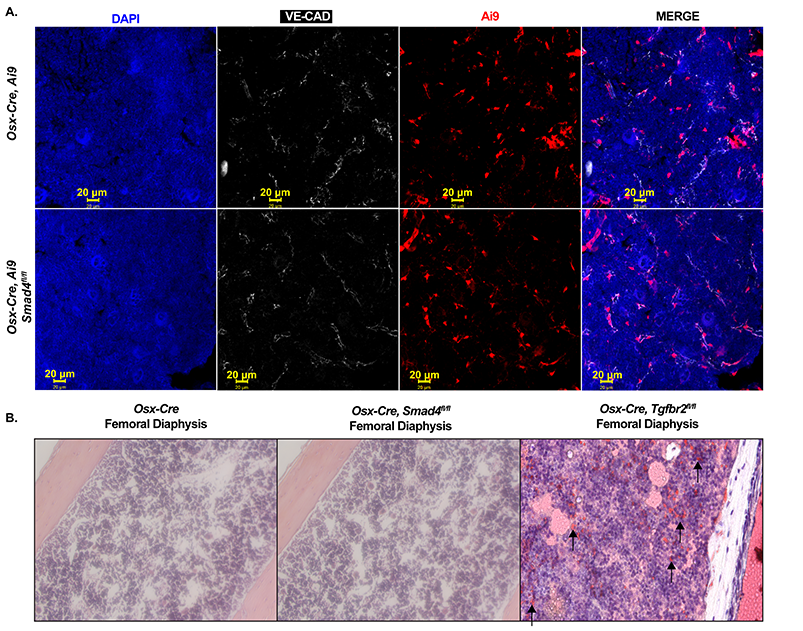

Supplement: S1 Fig — (A) Representative photomicrographs of femur sections from Osx-Cre Ai9 (control) or Osx-Cre, Ai9, Smad4fl/fl (Smad4fl/fl) mice showing DAPI stained nuclei and tdTomato (Ai9) mesenchymal stromal cells with morphologic similarities to CAR cells. (B) Representative images of femurs stained with oil red o (purple/red staining) to identify adipocytes (black arrowheads). Femur sections from Osx-Cre Tgfbr2fl/fl mice are included as a positive control, since we previously showed that constitutive deletion of Tgfbr2 in mesenchymal stromal cells is associated with a marked increase in bone marrow adiposity (Abou-Ezzi, Stem Cell Reports, 2019). (TIF) [file pone.0233751.s001.tif]

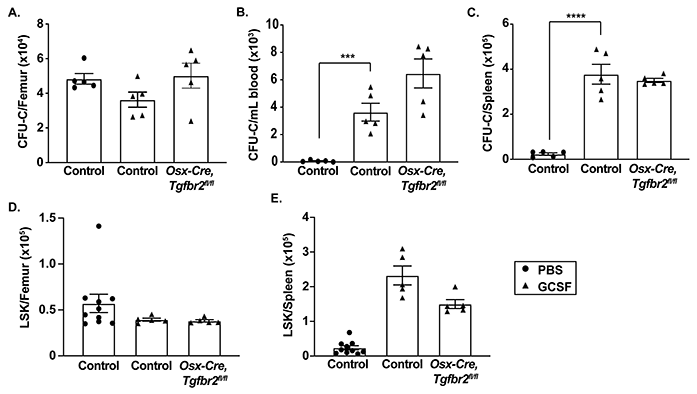

Supplement: S2 Fig — (A-C) Osx-Cre Tgfbr2fl/fl or Osx-Cre (control mice) were treated with 125 mg/kg of granulocyte-colony stimulating factor (G-CSF) twice daily for 7 days (). Shown are the number of colony forming cells (CFU-C) in bone marrow (A), blood (B) or spleen (C). D-E) Shown are the number of LSK cells in bone marrow (D) and spleen (E) after 7 days of G-CSF. Data represent the mean ± SEM. ***P < 0.001 and ****P < 0.0001 by two-way ANOVA with an alpha of 0.05 and Sidak’s multiple comparisons test. The saline treated cohort is the same as in Fig 5. (TIF) [file pone.0233751.s002.tif]
